# Temperature-induced surface reconstruction and interface structure evolution on ligament of nanoporous copper

**DOI:** 10.1038/s41598-017-18795-9

**Published:** 2018-01-11

**Authors:** Wenbo Liu, Peng Cheng, Jiazhen Yan, Ning Li, Sanqiang Shi, Shichao Zhang

**Affiliations:** 1 School of Manufacturing Science and Engineering, Sichuan University, Chengdu, 610065 China; 2 Department of Mechanical Engineering, The Hong Kong Polytechnic University, Hung Hom, Kowloon Hong Kong; 3 School of Materials Science and Engineering, Beihang University, Beijing, 100191 China

## Abstract

Micromorphology and atomic arrangement on ligament surface of nanoporous metals play a vital role in maintaining the structural stability, adjusting the reaction interface and endowing the functionality. Here we offer an instructive scientific understanding for temperature-induced surface reconstruction and interface structure evolution on ligament of nanoporous copper (NPC) based on systematically experimental observations and theoretical calculations. The results show that with dealloying temperature increasing, ligament surface micromorphology of NPC evolves from smooth to irregularity and to uniformly compressed semisphere, and finally to dispersed single-crystal nanoparticles accompanying with significant changes of interface structure from coherence to semi-coherence and to noncoherence. It can guide us to impart multifunctionality and enhanced reaction activity to porous materials just through surface self-modification of homogeneous atoms rather than external invasion of heteroatoms that may bring about unexpected ill effects, such as shortened operation life owing to poisoning.

## Introduction

Nowadays, nanoporous metals (NPMs) have attracted a lot of interest in a broad variety of applications involving actuators, catalysts, heat exchangers, microfluidic flow controllers, and so on^1–8^. Since it was found that dealloying technique can produce porous metals with a wide size range, during the past decade, much efforts has been dedicated to investigation of NPMs by dealloying^9–12^. Currently, two main aspects have attracted researchers’ focused attention. One is to synthesize NPMs with tunable microstructure and nanoporosity. Regular dealloying methods to prepare NPMs just can lead to formation of porous products with single pore size range, for example, 200 nm for NP-Cu^13^, 225 nm for NP-Ag^14^, and 13 nm for NP-Au^15^. In stark contrast, designing multi-step dealloying strategy can effectively achieve free-standing porous metals with multimodal pore size distributions typically by annealing-redealloying cycling treatment on Ag-plated NP-Au^16^. To minimize the manufacturing time and cost further, our group recently developed a series of facile one-step dealloying routes to mass preparation of porous metals with complicated porous architectures for promising industrialization, e.g. multiscale hierarchical structure^17–19^, asymmetrical structure^20,21^, and multi-layered sandwich structure^22,23^. The other aspect is surface modification of NPMs that can impart multifunctionality and enhanced properties to porous materials due to their monolithic morphology, unique interface structure, large specific surface area and excellent electrical conductivity. Typically, Pt-decorated NPMs markedly enhanced the catalytic activity as electrocatalysts for proton exchange membrane fuel cells and for glucose electrooxidation^24,25^. Sn/Ge-modified NPMs greatly improved the Li^+^ diffusion kinetics and cycling stability as anodes for Li-ion batteries^26,27^. Co_3_O_4_-embellished NPMs dramatically raised the detection sensitivity and accuracy as microelectrodes for electrochemical nonenzymatic glucose biosensors^28^. All these are closely related to unique porous architecture facilitating accommodation of volume change, increase of active site as well as fast infiltration of electrolyte and involved diffusion species. Obviously, micromorphology and atomic arrangement on ligament surface of NPMs can play a vital role in adjusting and controlling the structural stability and reaction kinetics of interface, such as deposition nucleation and growth, surface activity, bonding strength and functionality. To the best of our knowledge, however, few monographic studies on this issue have been made so far, which is considerably beneficial for profoundly understanding the intrinsic nature and unique characteristics (nanoporosity, interface activity and atom occupancy) of NPMs, but meanwhile is also a great challenge to us.

In this report, Al 30 at.% Cu alloy was taken as a typical instance to investigate the temperature-induced surface reconstruction and interface structure evolution on ligament of NPC. In the meantime, the surface diffusivity and diffusion activation energy are evaluated to reveal the intrinsic nature of formation of nanoporous structure, including initial pore size, critical characteristic length, shortest formation time, etc. Based upon our deep understanding of dealloying physical nature, evolution mechanism is discussed in detail.

## Results and Discussion

**Figure 1:**
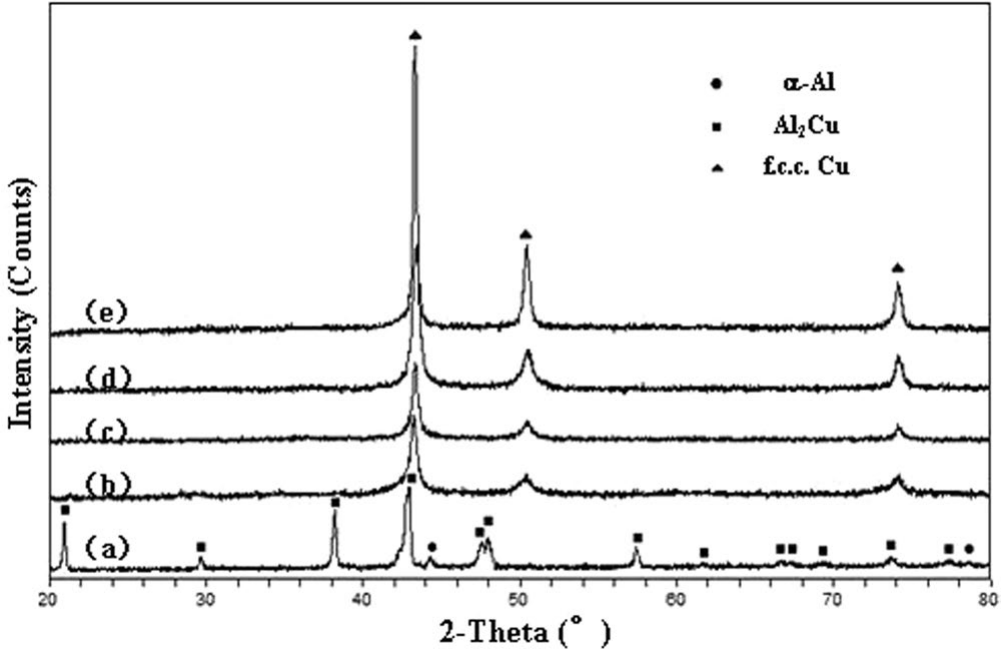
XRD patterns of melt-spun Al 30 at.% Cu alloy (**a**) before and (**b**–**e**) upon dealloying in the 5 wt.% HCl solution at 298 K, 333 K, 348 K and 363 K, respectively.

Figure 1 shows the XRD patterns of the initial Al 30 at.% Cu alloy ribbons and their as-dealloyed specimens after dealloying in the 5 wt.% HCl solution at different temperatures. The initial Al-Cu alloy consists of α-Al and Al_2_Cu phases, in which the amount of Al_2_Cu phase is greatly dominant in the alloy relative to α-Al. After the dealloying at different temperatures, just a face-centered cubic (f.c.c.) Cu phase can be determined in the resultant specimens, as displayed in Fig. 1b–e. Note that the peak width of (111)_Cu_, (200)_Cu_, (220)_Cu_ reflections of porous products by dealloying at RT is slightly broad compared to those at high temperatures, implying that the feature sizes of porous structure dealloyed at RT is relatively smaller^29^.

**Figure 2:**
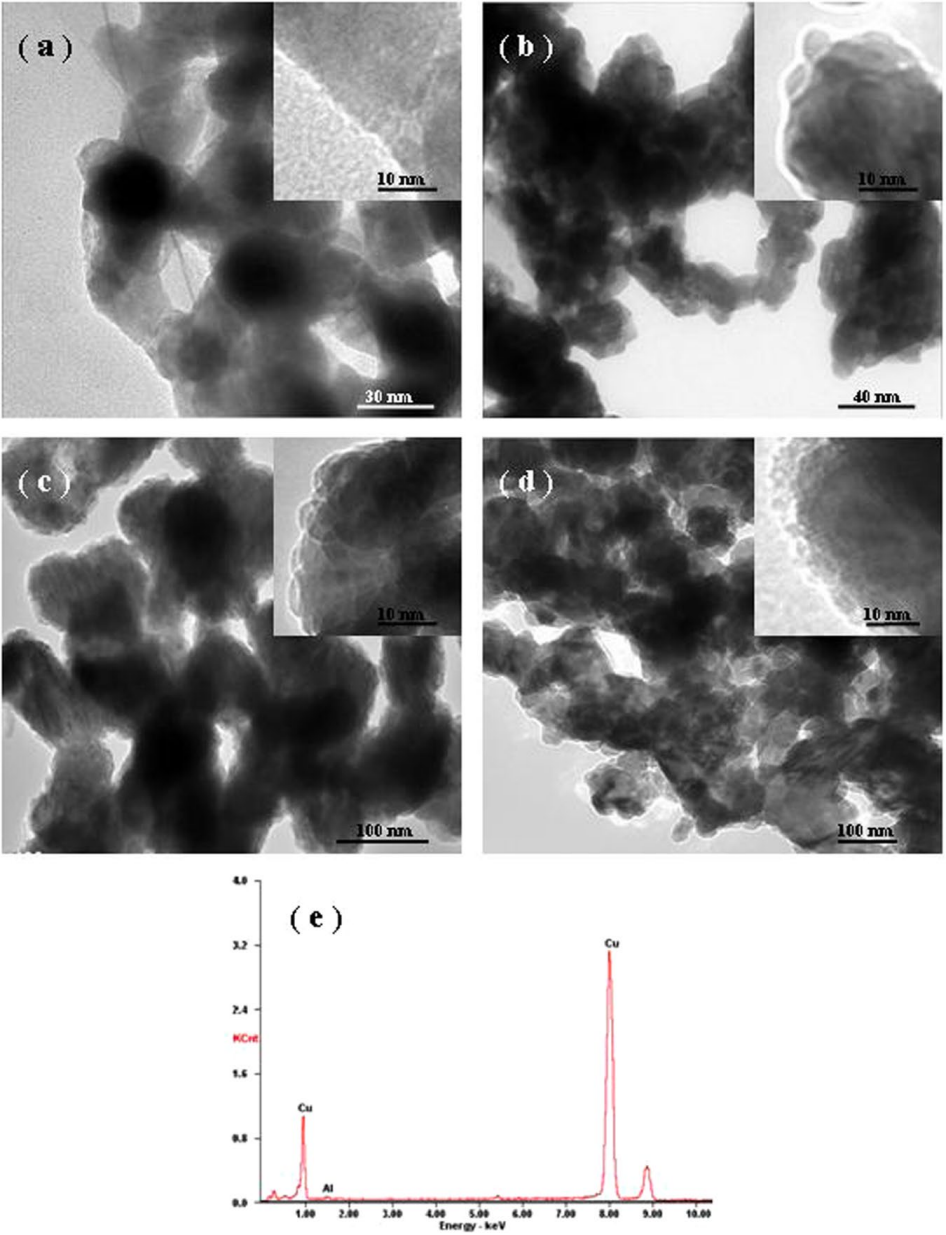
TEM images showing the ligament surface micromorphology of NPC by dealloying of the Al 30 at.% Cu alloy in the 5 wt.% HCl solution at (**a**) 298 K, (**b**) 333 K, (**c**) 348 K, and (**d**) 363 K, respectively. Insets show the corresponding elaborate micromorphology of ligament surface at a high magnification. (**e**) A typical EDX spectrum shows the chemical composition of the resultant NPC.

Figure 2 shows the TEM images of porous structure and surface micromorphology of NPC by dealloying of the Al-Cu alloy in the HCl solution at different temperatures. Obviously, an open, bicontinuous interpenetrating porous network with uniform pore size distributions can be achieved upon the dealloying at different temperatures, in which their ligament/pore sizes gradually increase from 30 ± 5 nm upto 150 ± 10 nm with increasing the dealloying temperatures ranging from 298 K to 363 K, as indicated in detail in Table 1. Meanwhile, note that the surface micromorphology generates an essential variation, as presented in the insets of Fig. 2. A smooth and straight ligament surface without obvious surface embossment can be clearly observed in the NPC dealloyed at RT. With increasing the dealloying temperature, the ligament surface of NPC gradually roughens and emerges irregular embossment with ~3 nm in height. As the dealloying temperature reaches 348 K, the irregular surface embossment can be replaced by uniformly compressed semispherical counterpart with ~4 nm in diameter. Finally, the superfine nanoparticles with a size of ~5 nm can be found on the ligament surface of NPC as the dealloying temperature climbs upto 363 K. These unique ligament surface micromorphology can be considered to be quite favorable for potential applications of photo/electrochemical catalysts because of more active sites exposed for photo/electrochemical reactions and shorter migration distance for ion transports. More importantly, we herein provide a facile approach to achieve the surface modification of homogeneous atoms as well as the adjusting and control of ligament surface micromorphology of NPMs through simple temperature change. In addition, EDX analysis has been carried out on the NPC dealloyed at different temperatures and one typical spectrum is displayed in Fig. 2e. Evidently, almost all of Al atoms was etched away from the initial alloy upon the dealloying. In stark contrast, nanoporous Au (by dealloying of Ag-Au solid solution alloys in a concentrated HNO_3_ solution) always involves some residual at.% Ag, which is anticipated to be trapped inside the gold ligaments in terms of the underlying physical mechanism of dealloying and normally just can asymptotically reach a limit at a long enough dealloying times (such as 100 h)^30,31^.

**Table 1:** The ligament sizes of NPC by dealloying of the Al-Cu alloy in the HCl solution at different temperatures, the corresponding surface diffusivities estimated by Eq. (1) ^42,43^, and the ratio of surface diffusivities at elevated temperatures (ET) to that at RT.

Dealloying temperature (T, K)	Dealloying time (t, s)	Ligament size (d(t), nm)	Surface diffusivity (D_S_, m^2^ s^−1^)	D_S_^ET^/D_S_^RT^
298	36000	30 ± 5	9.45 × 10^−20^	1
333	1800	50 ± 5	1.63 × 10^−17^	1.72 × 10^2^
348	1800	90 ± 10	1.79 × 10^−16^	1.89 × 10^3^
363	1800	150 ± 10	1.44 × 10^−15^	1.52 × 10^4^

**Figure 3:**
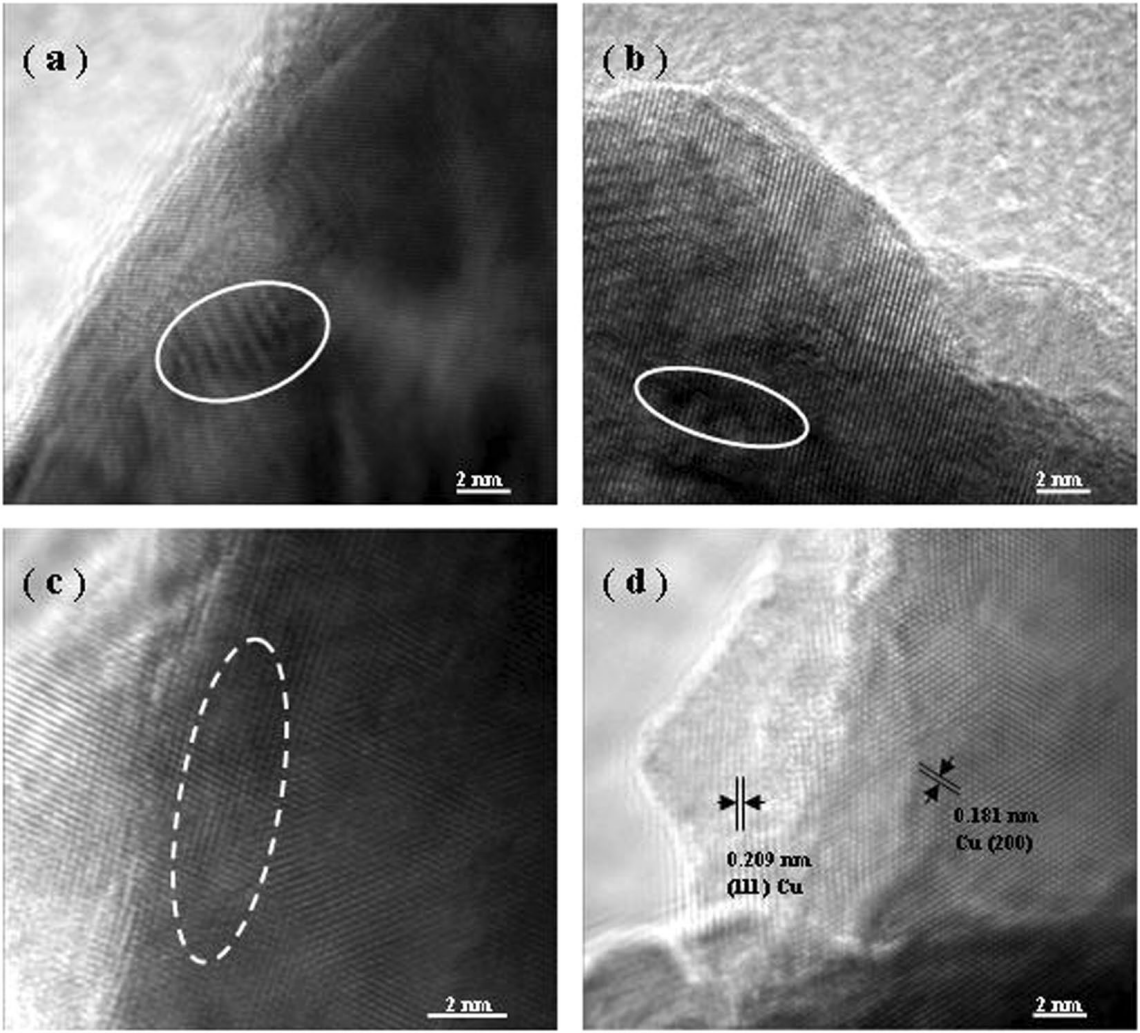
HRTEM images showing the interface structure and atomic arrangement in the ligament of NPC by dealloying of the Al 30 at.% Cu alloy in the 5 wt.% HCl solution at (**a**) 298 K, (**b**) 333 K, (**c**) 348 K, and (**d**) 363 K, respectively.

Figure 3 further shows the HRTEM images of interface structure between surface embossment and ligament matrix of NPC by dealloying at different temperatures. It is clear that just can a mild lattice distortion be seen on the ligament surface of NPC dealloyed at RT, as highlighted by solid ellipse in Fig. 3a, which is likely related to the surface stress during the etching^32^. When the dealloying temperature is 333 K, still no obvious difference in atomic arrangement of boundary between surface embossment and ligament matrix but more visible lattice distortions (marked by solid ellipse in Fig. 3b) can be found at this moment, as well as the lattice fringes can extend throughout the whole boundary, characteristic of typical coherent boundary originating from the epitaxial growth of Cu atoms on ligament surface of NPC through diffusion. However, it is worth noting that the present experimental results are substantially distinct from the established opinion that the crystal orientation can be reserved during the dealloying of solid solutions, such as Ag-Au, with conservation of grain dimension of initial alloys^33–36^. This is mainly because that the crystal structure of the resultant NPC is quite different from that of Al_2_Cu intermetallics in the initial alloy (Al_2_Cu: b.c.t; Cu: f.c.c.). With the temperature upto 348 K, the partial disorder of atomic arrangement on the boundary between surface embossment and ligament matrix can be observed clearly, as indicated by dashed ellipse in Fig. 3c, suggesting that it is typical of semi-coherent boundary probably resulting from the interface atomic rearrangement that minimizes the strain energy by high-temperature induced diffusion. As the dealloying temperature reaches as high as 363 K, the crossed lattice fringes with interplanar spacings of 0.209 nm and 0.181 nm from the HRTEM image in Fig. 3d, assigning to the (111) and (200) reflections of Cu respectively, manifest that the boundary is featured of noncoherent, which is closely associated with the recrystallization driven by greater mobility of Cu atoms at such an elevated temperature^37–39^. As a result, a clear boundary between Cu nanoparticle and ligament matrix can be distinguished in light of their apparently different lattice orientations. Note that the lattice fringes from Cu nanoparticle extending throughout the whole particle indicates its single crystal nature. This present experimental results further demonstrate that the ligaments of NPC are composed of polycrystalline copper rather than the monocrystals which is typically observed in the ligaments of NP-Au by dealloying of prototypical Ag-Au alloys^33^.

**Figure 4:**
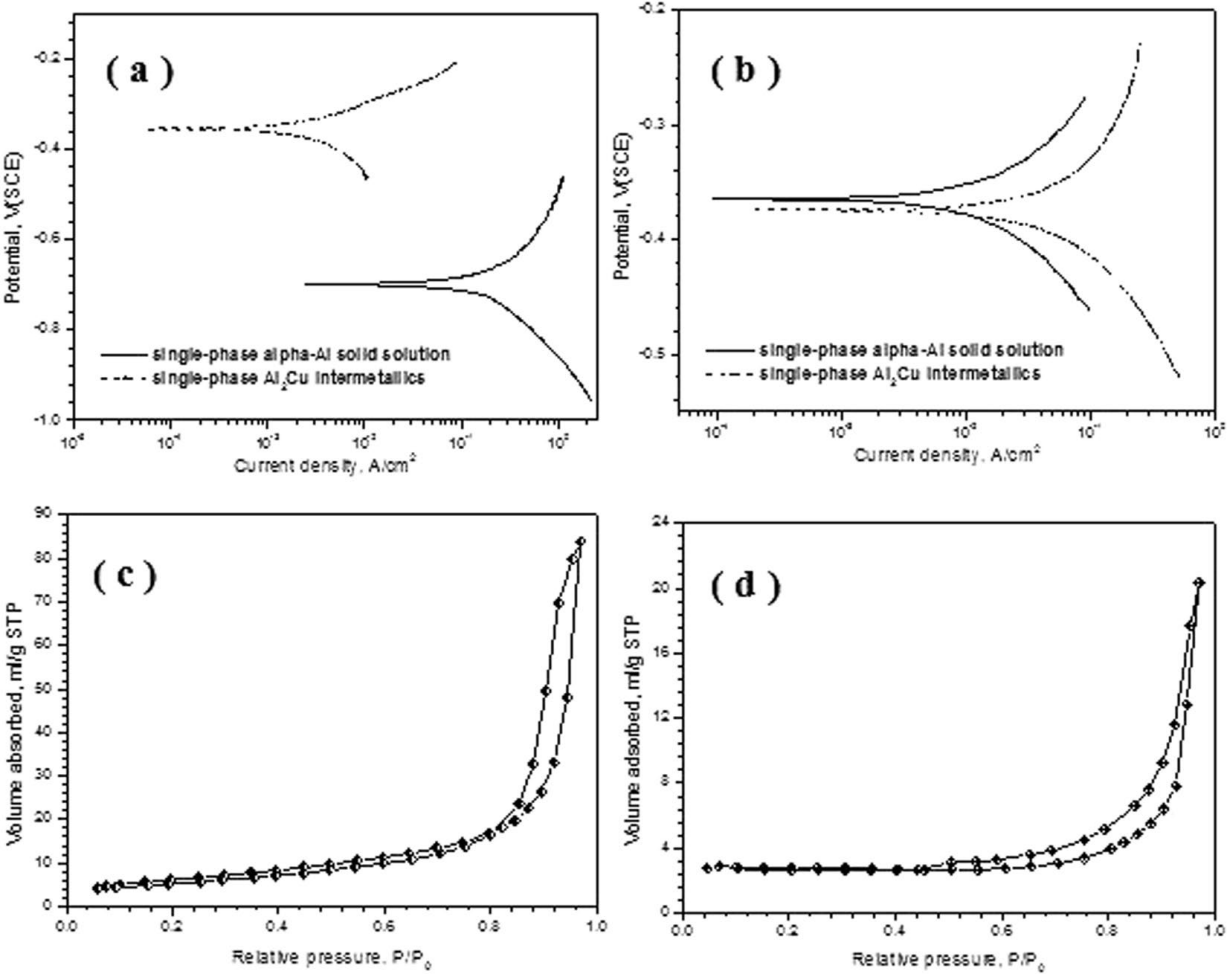
Tafel polarization curves of single-phase α-Al solid solution and Al_2_Cu intermetallics in the 5 wt.% HCl solution at (**a**) 298 K, and (**b**) 348 K. N_2_ isotherms at 77 K for the NPC by dealloying of the Al 30 at.% Cu alloy in the 5 wt.% HCl solution at (**c**) 298 K, and (**d**) 348 K.

Figure 4a,b shows Tafel curves of single α-Al and Al_2_Cu phases in the 5 wt.% HCl solution at different temperatures. It can be found that the disparity between corrosion potentials of α-Al and Al_2_Cu phases at 298 K is ~343.1 mV(SCE), considerably larger than that at 348 K (~9.91 mV(SCE)). The results explicitly indicate that α-Al and Al_2_Cu have the extremely closer electrochemical activities in the HCl solution at the elevated temperature, while a significantly higher activity can be acquired for α-Al solid solution at RT than Al_2_Cu intermetallics. Moreover, the specific surface areas of these NPC have been evaluated and their mesoporous feature has been further confirmed based upon N_2_ adsorption/desorption experiments, which shows a type IV isotherm with the H1 hysteresis loop. Figure 4c,d shows the N_2_ adsorption/desorption isotherms for NPC dealloyed at different temperatures. The Brunauer-Emmett-Teller (BET) surface areas of porous products are pretty high and have been identified to be 28.2 ± 0.1 and 13.6 ± 0.1 m^2^ g^−1^ for 298 K and 348 K, respectively. Obviously, the BET surface area at 298 K is two times larger than that at 348 K, which would be more beneficial for photo/electrochemical catalysis applications. Additionally, both the large BET surface area and forward-shifting hysteresis loop position of adsorption/desorption isotherm further demonstrate relatively small ligament/pore sizes of porous structure in the NPC dealloyed at RT, well consistent with the XRD analysis and TEM observations.

It is well-known that dealloying comprises dissolution of less noble (LN) elements and formation/coarsening of nanoporous structure by surface diffusion of more noble (MN) elements, in which surface diffusivity of MN atoms plays a crucial role in the formation/coarsening of NPMs and has a key impact on the ligament/pore feature sizes^9,40,41^. For thermal activation process, obviously, temperature is one of the most important factors to affect the surface diffusivity of MN elements. This is why the disparity among surface diffusivities is growing continually with the increase of dealloying temperature, which could be indicated by a parameter of ${{D}_{S}}^{ET}/{{D}_{S}}^{RT}$. ${{D}_{S}}^{ET}/{{D}_{S}}^{RT}$ refers to the ratio of surface diffusivity at an elevated temperature to that at RT, which would be discussed in detail on a basis of estimation of surface diffusivity and activation energy hereinafter.

A maximally unstable spatial period relationship can be predicted reportedly for feature size (*d*) and surface diffusivity (*D*_*S*_) of NPMs: $d\propto {({D}_{S}/{V}_{0})}^{\mu }$, where ${V}_{0}$ is the diffusion velocity of a plane alloy surface with no MN atoms aggregating on it and *μ* is a constant, proposed to be 1/4 or 1/6^9,42^. According to the coarsening mechanism controlled by surface diffusion, the ${D}_{S}$ of Cu atoms can be calculated by Eq. (1) as follows^42,43^:

1 $${D}_{S}=\frac{{[d(t)]}^{4}kT}{32\gamma t{\alpha }^{4}},$$

where $d(t)$ is the ligament feature size at dealloying time *t*, *k* is the Boltzmann constant (1.3806 × 10^−23^ J K^−1^), *T* is the dealloying temperature, *γ* is the surface energy (1.79 J m^−2^)^44^, and *α* is the lattice parameter of Cu (3.6153 × 10^−10^ m). In light of the parameters for dealloying at different temperatures in Table 1, the corresponding ${D}_{S}$ of Cu atoms can be obtained, also listed in Table 1. It can be found that ${{D}_{S}}^{ET}/{{D}_{S}}^{RT}$ significantly increases with increasing etching temperature from 298 K to 363 K, indicative of the evidently greater diffusion rate at elevated temperature than that at RT. It is astonishing that the ${D}_{S}$ of 1.44 × 10^−15 ^m^2^ s^−1^ at 363 K is four orders of magnitude greater than that of 9.45 × 10^−20^ m^2^ s^−1^ at RT, and a maximal ${{D}_{S}}^{ET}/{{D}_{S}}^{RT}$ value beyond 10000 can be reached. It is worth noting that in the previous literature by Erlebacher^40^, the ${D}_{S}$ value of Cu atoms has been reckoned theoretically to be 1.2 × 10^−14 ^m^2^ s^−1^ in vacuum and inferred to be of order 10^−10 ^m^2^ s^−1^ in electrolyte. Obviously, in contrast to our current results from experiments, that is an apparently overestimation, especially for the case at RT.

Generally, for most alloy systems suitable for dealloying, it is quite hard to obtain the initial ligament/pore feature sizes during etching since the nanoporous structure is very unstable and often undergoes rapid coarsening^45^. According to the kinetic process of formation/coarsening of NPC in this study, the initial ligament/pore feature sizes could be estimated for dealloying at different temperatures. In light of Eq. (1), [d(*t*)]^4^ is proportional to the etching time *t* at a given temperature. As a result, a ligament/pore size of ~3.9 nm, ~5.7 nm, ~6.8 nm, ~12.2 nm, ~21.7 nm can be predicted in NPC dealloyed in the HCl solution at 298 K for 10 s, 50 s, 100 s, 1000 s, 10000 s, respectively. In terms of the model of Sieradzki and co-workers^10^, there is a fixed feature size, *ζ* ^*perc*^, which is closely related to nanoclusters comprising interconnected LN atoms. For a binary alloy system, *ζ* ^*perc*^ can be acquired by the following equation:

2 $${\zeta }^{perc}={\alpha }_{nn}(1+p)(1-p),$$

where α_nn_ is the nearest-neighbour interval and *p* is the mole fraction of LN elements, respectively^10^. The initial pore size, *d*_0_, can be approximately given by 2*ζ* ^[ *perc*45^. In this case, *d*_0_ is estimated to be ~4 nm. Thus, the results clearly demonstrate that the formation of initial nanoporous structure needs ~10 s at least by dealloying of the Al-Cu alloy in the HCl solution at 298 K. In contrast, for the dealloying at 363 K, the ligament/pore feature sizes of NPC can be predicted by the same way as ~4.3 nm, ~7.6 nm, ~13.6 nm, ~24.2 nm, ~43.1 nm for 0.001 s, 0.01 s, 0.1 s, 1 s, 10 s, respectively. Intriguingly, an ultrahigh formation rate of initial nanoporous structure can be achieved at the elevated temperature, 10^4^ times faster than that at RT. This just is in good coincidence with the estimated ${{D}_{S}}^{ET}/{{D}_{S}}^{RT}$ value above.

**Figure 5:**
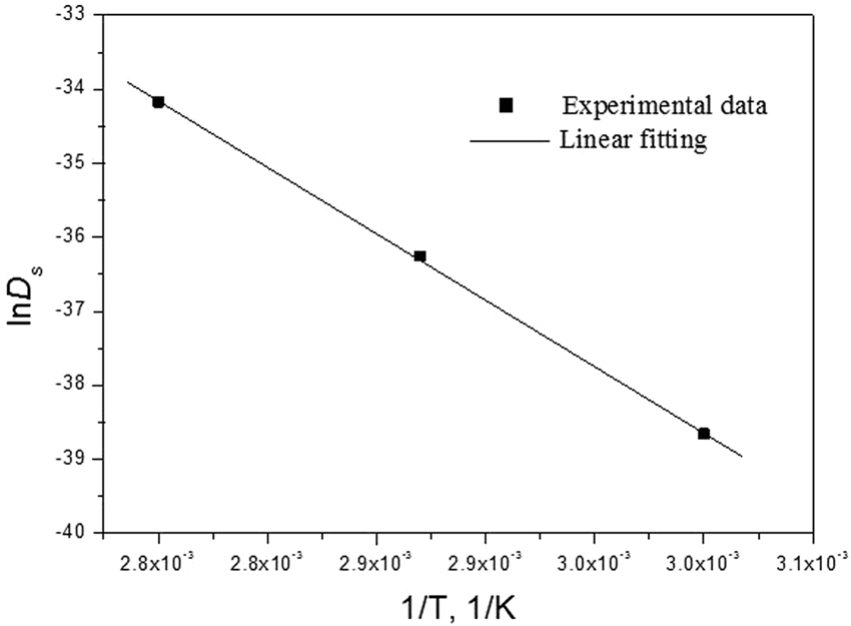
Plot of $\mathrm{ln}\,{D}_{S}$ vs. $1/T$ for the estimation of activation energy.

It has been reported that the Arrhenius equation for surface diffusion process is as follows^46^:

3 $${D}_{S}={D}_{0}\,\exp (-\frac{{E}_{a}}{RT}),$$

where ${D}_{0}$ is the pre-exponential factor, ${E}_{\alpha }$ is the diffusion activation energy, and $R$ is the gas constant (8.314 J mol^−1^ K^−1^). The measurement of diffusion activation energy for nanoporous structure formation would be in favour of understanding the underlying physical mechanism of dealloying due to its thermal activation nature^42^. Figure 5 shows the plot of $\mathrm{ln}\,{D}_{S}$ vs. $1/T$ tested at different temperatures. It is clear that a good linear relationship can be gained for the experimental data of $\mathrm{ln}{D}_{S}$. In terms of the intercept ($\mathrm{ln}{D}_{0}$) and the slope ($-{E}_{\alpha }/R$), ${D}_{0}$ and ${E}_{\alpha }$ can be determined accordingly. It can be found that the ${E}_{\alpha }$ is ~63.2 kJ mol^−1^ in this case, quite approximate to the data in literature^47^, strongly implying that the formation/coarsening of NPC depends upon the diffusivity of Cu atoms along alloy/solution interfaces regardless of etching solution species. Moreover, the present ${E}_{\alpha }$ value is far lower than activation energy of surface diffusion of Cu atoms under vacuum (113 ± 10 kJ mol^−1^)^48^, which can be rationalized below: (1) Cu atoms interacting with solvent molecules/ions in the solution can cause the surface layer relaxation, resulting in the sharp descent of ${E}_{\alpha }$ ^49^; (2) the interactions between partially emptied orbitals of Cu atoms and sp^3^ orbitals of H_2_O molecules can lead to the metallic feature loss of Cu atoms^50^; (3) the rapid increase of local concentration of Cu atoms after Al dissolution, ~30% beyond their equilibrium density of 10^−7^ per site^9^. As a result, there exists a considerable large driving force for Cu atoms to diffuse along alloy/solution interfaces. On the other hand, the diffusion constant (${D}_{0}$) of Cu atoms in the Al-Cu system is 5.48 × 10^−7^ m^2^ s^−1^, several orders of magnitude greater than that of Au atoms (~10^−11^ m^2^ s^−1^) in prototypical Ag-Au system^40^. This implies that there may be distinctive dealloying physical mechanism for intermetallics in contrast to solid solutions such as Ag-Au system. The dealloying physical mechanism of solid solutions has so far been elucidated well based on the disordering-reordering model^51^, the roughening transition model^52^, and the Monte Carlo model^9,40^. Typically, Parida and co-workers reported that the dealloying process of Ag-Au solid solution alloys would not need nucleation of new crystallites or formation/removal of lattice sites^35^. But it can be perceived that the formation and removal of lattice sites probably should be indispensable during the dealloying of Al-Cu intermetallics, such as Al_2_Cu. The underlying dealloying physical mechanism needs to be probed further, which would have great inspirations for understanding of nanoporosity evolution and dealloying behavior of intermetallics. Additionally, from the standpoint of thermodynamics, interface structure evolution from coherence to semi-coherence and to noncoherece can be well explained as a consequence of the intercompetition between interfacial energy and configurational energy to make the system being more stable with lower energy state. Based on our present findings, the facile achievement of adjusting and control of surface micro-surroundings and interface atomic arrangement may impart multifunctionality and enhanced reaction property to porous materials due to the more active sites and faster ion/electron transport channels. To the best of our knowledge, it is first to offer an instructive scientific understanding for surface reconstruction and interface structure evolution on ligament of NPMs by facile temperature control.

## Conclusions

To summarize, temperature-induced surface reconstruction and interface structure evolution on ligament of NPC have been investigated systematically based on experimental observations and theoretical calculations. With the increase of dealloying temperature, ligament surface micromorphology of NPC evolves from smooth to irregularity and to uniformly compressed semisphere, and finally to dispersed superfine single-crystal nanoparticles accompanying with significant changes of interface structure from coherence to semi-coherence and to noncoherence, which can be explained as a consequence of the intercompetition between interfacial energy and configurational energy to make the system being more stable with lower energy state. This present work will have important implications for imparting multifunctionality and enhanced reaction activity to porous materials just through surface self-modification of homogeneous atoms rather than external invasion of heteroatoms that may bring about unexpected ill effects.

## Methods

### Synthesis of initial Al-Cu precursor and resultant NPC

Al-Cu alloy with 30 at.%_Cu_ was fabricated by pure Al and Cu (>99.99 wt.%). Arc heating was employed to melt the pure metals in a crucible under Ar atmosphere, and then the melt was poured into ingots. By using a melt spinning equipment, the Al-Cu alloy was remelted by induction heating and then melt-spun onto a copper roller at a rotating speed of ~2300 rpm. The thickness, width and length of as-made ribbons were 20–50 µm, 5–8 mm and 8–10 cm, respectively. Their chemical composition and element distribution were analyzed by Energy dispersive X-ray (EDX), as presented in Table S1 and Figure S1 (Supplementary Information), which is very closely to the designed composition, indicating their availability in the following study. Subsequently, the dealloying of the Al-Cu ribbons was performed in a 5 wt.% HCl aqueous solution at temperatures ranging from room temperature (RT, 298 K) to 363 K until no bubbles emerged. The typical treatment duration was ~ 10 h at RT and ~ 30 min at elevated temperatures, respectively. Upon the dealloying, the resultant products were rinsed by using distilled water and dehydrated alcohol several times. The as-dealloyed products were kept in a vacuum chamber to avoid oxidation.

### Microstructure and phase characterization

Microstructure, phase characterization and analysis of samples before and after the dealloying were carried out by X-ray diffraction (XRD, Rigaku D/Max-2400) with Cu K_α_ radiation, transmission electron microscopy (TEM, JEOL JEM 2100F) with an EDX analyzer and high-resolution transmission electron microscopy (HRTEM, JEOL JEM 2100F).

### Assessment of electrochemical activity and specific surface area

In order to measure the activities of α-Al and Al_2_Cu phases in the Al-Cu alloy, potentiodynamic polarization curves were tested on single α-Al solid solution and Al_2_Cu intermetallics in the HCl solution at different temperatures by use of electrochemical workstation (PARSTAT 2273). The test was conducted in a standard three-electrode system (200 mL) with Pt wire electrode as counter electrode, saturated calomel electrode (SCE) as reference electrode, and the test sample as working electrode. Polarization scan was performed towards positive direction at a scan rate of 1.0 mV s^−1^ after reaching a steady state potential. To further assess surface area of products, the N_2_ adsorption/desorption profiles were monitored at 77 K on a Nova Station A automatic surface area and pore size distribution instrument.

## Electronic supplementary material

Supplementary Information
