# Supplementary material for: Temperature-induced surface reconstruction and interface structure evolution on ligament of nanoporous copper
Source: Sci Rep. 2018 Jan 11;8:447. doi: 10.1038/s41598-017-18795-9 (PMC5765166; doi:10.1038/s41598-017-18795-9)
Supplement: Supplementary file 1 — Supplementary Information [file 41598_2017_18795_MOESM1_ESM.doc]

**Temperature-induced s****urface reconstruction and interface structure evolution on ligament of nanoporous copper**

Wenbo Liu,^a,b,*^ Peng Cheng,^a^ Jiazhen Yan,^a^ Ning Li,^a^ Sanqiang Shi,^b^ Shichao Zhang^c^

^a^ School of Manufacturing Science and Engineering, Sichuan University, Chengdu 610065, China

^b^ Department of Mechanical Engineering, The Hong Kong Polytechnic University,

Hung Hom, Kowloon, Hong Kong

^c^ School of Materials Science and Engineering, Beihang University, Beijing 100191, China

Tel: +86-028-85405320; Fax: +86-028-85403408; E-mail: liuwenbo_8338@163.com.

**Table S1.** Chemical composition of the original Al-Cu ribbons by EDX analysis.

| Specimens | Elements (at.%) | |
| --- | --- | --- |
|  | Al | Cu |
| Original Al-Cu ribbons | 69.58 | 30.42 |


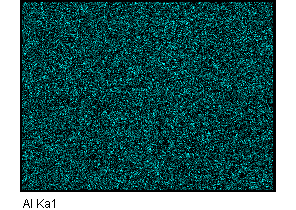

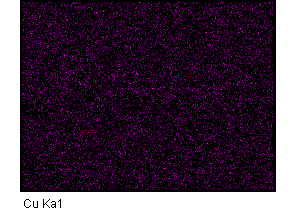

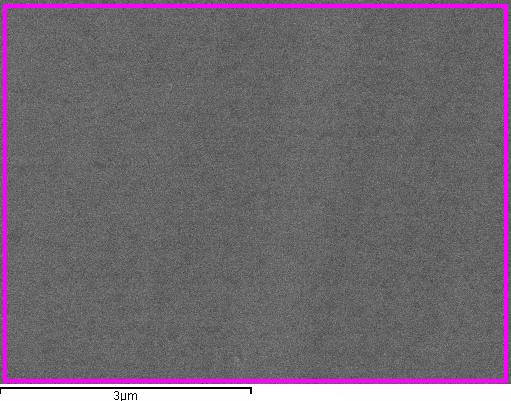


**1.5 um**

**Al Kα1**

**Cu Kα1**

**( a )**

**( b )**

**( c )**

**Figure S1.** The Al and Cu element mapping in the original Al-Cu alloy ribbons.

As can been seen from results of Table S1 and Figure S1, the Al and Cu element distribution is uniform in the original alloy and their chemical composition is quite closely to the designed alloy composition, indicating that the prepared alloy ribbons can be further used in the following study of this work.
